# Supplementary material for: Survival outcome and prognostic factors of patients with nasopharyngeal cancer in Yogyakarta, Indonesia: A hospital-based retrospective study
Source: PLoS One. 2021 Feb 12;16(2):e0246638. doi: 10.1371/journal.pone.0246638 (PMC7880494; doi:10.1371/journal.pone.0246638)
Supplement: S1 Table — (DOCX) [file pone.0246638.s001.docx]

**S1 Table. Distribution of education status by living areas**

| **Variable** | **Rural (n/%)** | **Urban (n/%)** | **Missing (n/%)** | **p** |
| --- | --- | --- | --- | --- |
| ≤9 years | 174 (47.7) | 71 (18.0) | 0 (0.0) | < 0.01 |
| >9 years | 136 (37.3) | 256 (64.6) | 2 (40.0) |  |
| Missing | 55 (15.0) | 69 (17.4) | 3 (60.0) |  |
