# Supplementary material for: Survival outcome and prognostic factors of patients with nasopharyngeal cancer in Yogyakarta, Indonesia: A hospital-based retrospective study
Source: PLoS One. 2021 Feb 12;16(2):e0246638. doi: 10.1371/journal.pone.0246638 (PMC7880494; doi:10.1371/journal.pone.0246638)
Supplement: S2 Table — (DOCX) [file pone.0246638.s002.docx]

**S2 Table. Mean BMI grouped by NPC staging**

| **NPC staging** | **Observations** | **Mean BMI** |
| --- | --- | --- |
| I - II | 50 | 22.4 |
| III - IVB | 540 | 20.6 |
| IVC | 111 | 19.4 |

BMI: body mass index

NPC: nasopharyngeal cancer
