# Supplementary material for: Survival outcome and prognostic factors of patients with nasopharyngeal cancer in Yogyakarta, Indonesia: A hospital-based retrospective study
Source: PLoS One. 2021 Feb 12;16(2):e0246638. doi: 10.1371/journal.pone.0246638 (PMC7880494; doi:10.1371/journal.pone.0246638)
Supplement: S1 File — (PDF) [file pone.0246638.s004.pdf]

291O Survival outcome and prognostic factors of patients with nasopharyngeal cancer in Yogyakarta Indonesia

S.H. Hutajulu[1], D. Howdon[2], K.W. Taroeno Hariadi[1], M.S. Hardianti[1], I. Purwanto[1], S.R. Indrasari[3], C. Herdini[3], B. Hariwiyanto[3], A. Ghozali[4], H. Kusumo[5], W. Dhamiyati[5], S.R.D. Danarti[5], I.B. Tan[6], J. Kurnianda[1], M.J. Allsop[2]

[1]Division of Hematology and Medical Oncology, Department of Internal Medicine, Gadjah Mada University/Dr. Sardjito General Hospital, Yogyakarta, Indonesia, [2]Leeds Institute of Health Sciences, School of Medicine, Faculty of Medicine and Health, University of Leeds, Leeds, UK, [3]Department of Otorhinolaryngology Head and Neck Surgery, Faculty of Medicine, Public Health and Nursing, Gadjah Mada University/Dr. Sardjito General Hospital, Yogyakarta, Indonesia, [4]Department of Anatomical Pathology, Faculty of Medicine, Public Health and Nursing, Gadjah Mada University/Dr. Sardjito General Hospital, Yogyakarta, Indonesia, [5]Department of Radiology, Faculty of Medicine, Public Health and Nursing, Gadjah Mada University/Dr. Sardjito General Hospital, Yogyakarta, Indonesia, [6]Department of Otorhinolaryngology Head and Neck Surgery, Maastricht University Medical Center, Maastricht, Netherlands

Background: The survival of patients with nasopharyngeal cancer (NPC) have been reported in endemic and non-endemic regions. However, this has never been analyzed in Indonesian cases although this malignancy is endemic. This study aimed to perform survival analysis of the local cases and determine the prognostic factors.

Methods: A total of 767 patients with NPC diagnosed from January 2007 to December 2016 at Dr Sardjito General Hospital were included. Potential prognostic variables included sociodemographic parameters, clinicopathology features and treatment strategy. Kaplan-Meier method and the log-rank test estimated the rates of overall survival (OS). Multivariate analyses were done by a Cox proportional hazards model.

Results: The median OS was 14.38 months for the whole cohort. Patients with education $\geq$9 years, government insurance for civil servants, advanced stage and combination of chemo-radiation treatment had a significantly better OS than their counterparts ($p < 0.005$, $p < 0.005$, $p < 0.050$ and $p < 0.001$). Multivariate analyses showed that age, clinical stage and treatment modality were independent prognostic factors for OS. For age, individuals over the median age had a reduced OS (time ratio/TR= 0.733, 95% confidence interval/CI 0.570-0.944). Individuals with advanced clinical stage (stage IVc) also had a reduced OS (TR = 0.123, 95% CI 0.21-0.737). In stratified analysis, compared with chemotherapy or radiation alone, chemo-radiation demonstrated a significant benefit of the patients' OS (TR 4.87; 95% CI 3.01-7.87).

Conclusions: Median OS of our cohort was low compared to those reported in international publications, emphasizing the importance to improve the local cancer management. Age, clinical stage and treatment modality were independent favourable predictos for the patients' OS.

Legal entity responsible for the study: Ethics committee, Faculty of Medicine, Public Health and Nursing Universitas Gadjah Mada/Dr Sardjito Hospital.

Funding: Indonesian Society for Hematology and Medical Oncology Yogyakarta Branch; Dutch Cancer Society.

Disclosure: All authors have declared no conflicts of interest.
